# Using a Model of Germ-Free Animals to Study the Impact of Gut Microbiome in Research: A Step by Step Sterility Setting and Management

**DOI:** 10.3390/mps3010018

**Published:** 2020-02-22

**Authors:** Odile Gabay, Jonathan Vicenty, Dylan Smith, Linda Tiffany, Jill Ascher, Tina Curry, John Dennis, Kathleen A. Clouse

**Affiliations:** 1 U.S. Food and Drug Administration, Center for Drug Evaluation and Research, Office of Biotechnology Products, Division of Biotechnology Review and Research 1, Silver Spring, MD 20993, USA; jdvg5064@gmail.com (J.V.); Dylan.Smith@fda.hhs.gov (D.S.); Linda.Tiffany@fda.hhs.gov (L.T.); Kathleen.Clouse@fda.hhs.gov (K.A.C.); 2 U.S. Food and Drug Administration, Center for Biologics Evaluation and Research, Office of Management, Division of Veterinary Services, Silver Spring, MD 20993, USA; Jill.Ascher2@nih.gov (J.A.); Tina.Curry@fda.hhs.gov (T.C.); John.Dennis@fda.hhs.gov (J.D.)

**Keywords:** microbiome, germ free, sterility method, animal model

## Abstract

The particularly unique composition of the gut microbiota has the potential to influence the health or disease status of animal and human hosts. Altering the homeostasis of the host-bacteria could lead to changes in gut flora that result in disease or activation of a specific immunological response, which could explain the variations observed in patient responses to current therapies. A standardized model is crucial for studying the influence of the gut microbiota on therapeutic modalities. A step by step mouse model and sterility management system that compares a control strain of C57BL/6 mice to the established C57BL/6 germ-free (GF) strain has been developed. The GF BL/6 mouse phenotype is well established, and the anatomical differences between the GF and control mice were evident in this model. This method could be applied to research studies investigating the microbiome impact, the response to various therapies, or disease transfer via fecal transplants. A standardized sterility maintenance method is crucial in this context.

## 1. Introduction

The gut microbiome continues to be a focus of intense research. A recent publication catalogs the mouse gut metagenome, which is similar to that in humans, and revealed that approximately 99% of cataloged genes are bacterial. Furthermore, 95.2% of cataloged bacterial genes have been identified for humans and mice [1] The remaining 1% consists of viruses, fungi, worms, and other resident microorganisms [2]. Clinical data provide strong evidence that a stable and diverse gut microbial composition is important for the maintenance of optimal health [3,4]. Altered microbial profiles, resulting in altered homeostasis of the host-bacteria, have been associated with numerous human illnesses [5,6,7].

To assess the critical question of whether commensal bacteria play a role in a given disease and/or contribute to therapy failure, the ideal model would use germ-free (GF) animals, totally devoid of bacteria, in parallel with wild-type animals having a defined microbiota. Although use of an animal model to address this question is not ideal, designing such a model in humans is not ethically possible. Currently, germ-free studies most commonly use mice [8]. However, rats (Sprague Dawley), chickens and guinea pigs have been used, with piglets and calves used less extensively [8,9].GF mouse strains have been maintained continuously since 1954. Methods of obtaining GF animals were simplified by the development and improvement of isolation equipment [10]. Today, two distinct methods for obtaining GF mice exist: aseptic cesarean or embryo transfer, thereby reducing the risk of vertical contamination. The challenge, however, is to maintain a sterility barrier to avoid contamination, which is common in laboratories, and therefore introduce study bias. To our knowledge, no detailed method describing a protocol have been published in the literature. 

The purpose of this preliminary work was to establish a protocol for comparing the response to a therapeutic agent under controlled (germ-free) conditions to settings in which the characteristic microbiota (wild-type) is maintained. A detailed method, easy to follow step by step, is proposed to maintain a continuous sterility flow thereby avoiding sterility breaches and highlighting the importance of not disrupting the microbiome of mice included in therapeutic cohorts (germ-free, gnotobiotic, or conventional controls) by the addition of antibiotics or using a method of sterilization impacting food nutrients, thereby introducing bias into the study. The impact of the microbiome on the variability of responses to a chosen therapeutic agent could then be investigated. Maintenance of a sterile environment without breach is crucial. A similar approach could be used for fecal transplants in GF animals, rendering them gnotobiotic for disease transmission studies. 

## 2. Experimental Design

The first challenge faced when setting up a model using a germ-free colony was to ensure that the mice were housed in an environment totally free of pathogens, including viruses, fungi, and bacteria, and that non-germ-free controls were housed under the same conditions with respect to bedding, food and water supplies. 

### Materials and Equipment

**Figure 1:**
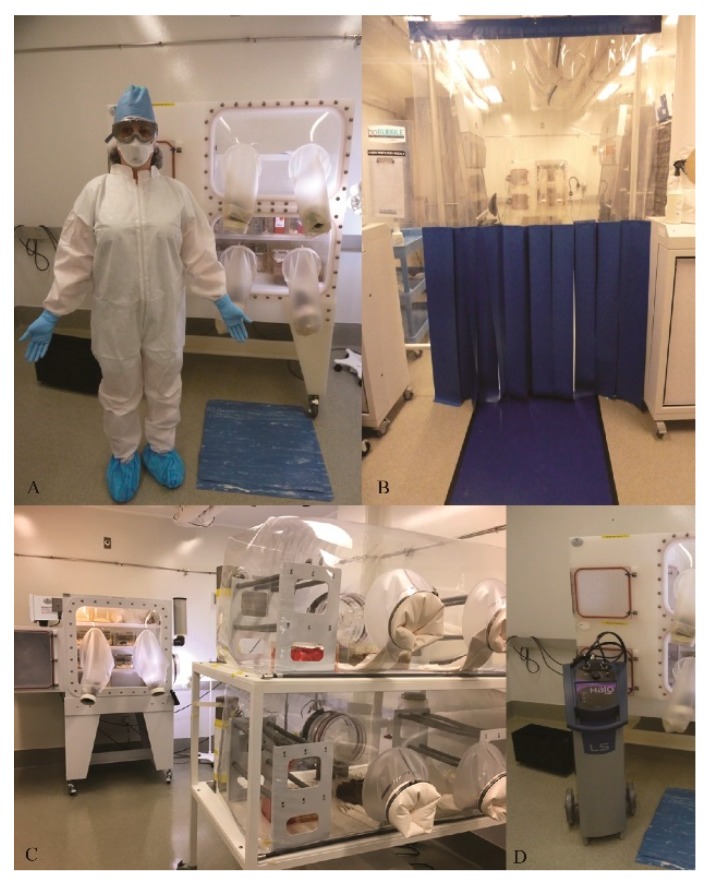
Equipment of the sterile environment (Bio-Bubble). (**a**) Personal Protective Equipment (PPE) required while using the Bio-Bubble: disposable coveralls, double shoe covers, double gloves, mask, double hair covers, goggles. (**b**) Antechamber dedicated to decontamination: sterile curtains separating antechamber and sterile room containing isolators and hood. (**c**) Sterile semirigid isolator, SRI, and flexible isolator. On the left of the SRI, port of entry, with sterile chamber for introduction of supplies. (**d**) Fogger (Quip) used to spray decontaminant inside the isolators and in the room prior to use.

- A devoted room and technical team are basic requirements. GF mice can be housed in sterile semirigid isolators (SRI) or in flexible isolators (Park Bioservices, LLC, Groveland, MA, USA) (Figure 1C). We chose to work with SRI. Each isolator, depending on its size, can hold up to 12 cages, each housing four mice.
- C57 BL/6 GF mice, as well as their control C57BL/6 littermates, can be purchased from several authorized vendors, as well as other germ-free strains such as BALB/c, Swiss Webster, or strains bred in-house. However, the latter poses a challenge due to the poor reproductive performance of the mice, attributable to restricted abdominal space from an enlarged cecum. In our studies, mice were purchased from Taconic (Taconic Bioscience, Rensselaer, NY, USA).
- All animal procedures and Animal Study Proposals (ASP) were reviewed and approved by an Institutional Animal Care and Use Committee (FDA White Oak IACUC for our studies).

## 3. Procedure

### 3.1. Setting up a Sterile Environment: (Figure 1)

#### 3.1.1. Set up and Preparation

The key to conducting a germ-free study is to ensure total sterility of the germ-free mouse environment. To maintain such an environment, a strict protocol must be established before initiating the study and it must be maintained for the duration of this study. 

Mice should be housed in a restricted-access room, accessible only to authorized personnel having proper training in germ-free handling (Figure 1B). This room should be prepared prior to housing the animals. A thorough decontamination of the room containing the isolators is first performed using a hydrogen peroxide (H_2_*O*_2_) vapor solution delivered by a fogger (HaloFogger Extended Nozzle, Quip Laboratories, Wilmington, DE, USA; Figure 1D).

Biological indicators (BI, Apex Biological Indicator for gaseous hydrogen peroxide) are placed prior to the fogging, and subsequently analyzed to verify adequate killing and determine bacterial log reduction. Specific personal protective equipment (PPE), which includes disposable coveralls, cap, mask, nitrile gloves, and shoe covers, is required to enter the room. Jewelry and perfume must be removed prior to putting on PPE. Use of a second set of shoe covers, cap and nitrile gloves are recommended prior to entering the GF room, as per specific protocol and Standard Operating Procedures (SOPs) (Figure 1A)

#### 3.1.2. Experimental Design

Before animals are ordered, ensure that all the supplies needed to conduct the study from inception to completion, including backups, are on hand. Isolators should be set up with all necessary sterile supplies needed to carry out the approved experiment, (i.e.; autoclaved cloth gloves, bags, forceps, paper towels, ear punch, labeled tubes, plastic rack, syringes and needles, scalpels, and disposable sharp mini-trash), prior to receipt and occupation by GF animals. This also includes preparation and autoclaving of the cages with fresh bedding (BioFresh Comfort White Auto, Lab Diet, Richmond, IN, USA). Bedding and environmental enrichment (Enviro-dri, Certified LabDiet, Richmond, IN, USA) are placed in the cage prior to autoclaving. Autoclaved, irradiated rodent chow and non-chlorinated water should also be prepared (Certified LabDiet, Richmond, IN, USA). Dechlorination of water is achieved by leaving water in an open container overnight so that chlorine in the water will dissipate prior to transfer into the water bottles for autoclaving. No antibiotics were added in the water, or in any other process to avoid bias in the microbiome studies. All materials entering the isolators were individually sterile wrapped, autoclaved, or appropriately sprayed with an approved sterilant (200 ppm MB-10, prepared with MB-10 tablets: 20.8% sodium chlorite, 7% sodium dichloroisocyanurate dihydrate solution; Quip Laboratories). A specific procedure used for transferring supplies into the isolator via a sterile port with 45 minutes’ wait time was implemented to insure sterilization. Careful planning helped minimize the number of material transfers into the isolator.

### 3.2. Working in the Sterile Environment (Figure 2)

#### 3.2.1. Procedure: Introduction of the Mice

**Figure 2:**
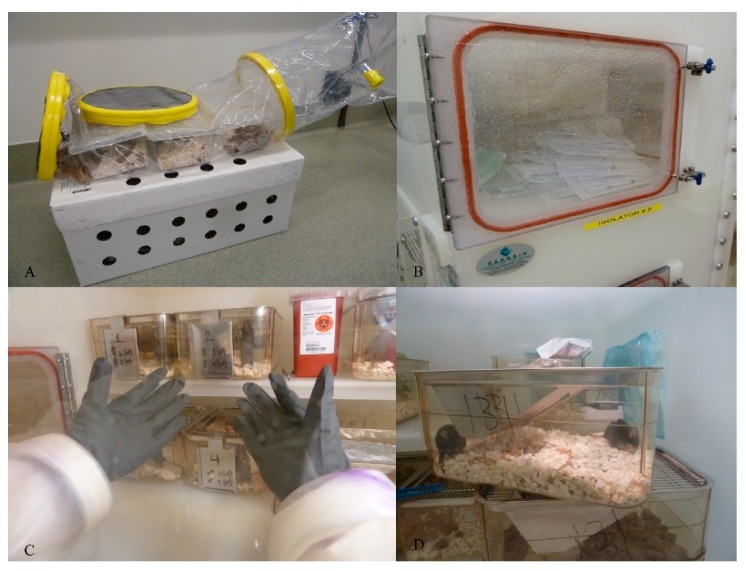
Sterility flow. (**a**) Shipment box used to deliver mice in a sterile environment. Flexible boxes are sealed with layers of tape and contain up to three cages. (**b**) Sterile port, with a hermetic door. Specific protocols outline procedures for introduction of supplies. (**c**) Interior gloves worn on top of sterile latex gloves to manipulate mice. (**d**) A cage with germ-free C57 BL/6 mice, containing specific autoclaved bedding, paper, food, and water.

The first step consists of appropriately housing the sterile animals immediately upon arrival. Transport boxes arrive from the vendor in cylinders sealed with tape, with separate sets of cages inside (Figure 2A). Two technicians are needed to successfully house the GF animals. The transport container is first sprayed with sterilant and placed directly in front of the isolator port entrance. A direct fit must be ensured between the port cylinder and the transport cylinder and the two parts are liberally sprayed with sterilant to complete saturation. The soft plastic portion of the cylinder is attached to the SRI port with multiple layers of nonporous packing tape and allowed to saturate for one hour. At this time, a health assessment of the GF animals is conducted by an animal health technician dedicated to the GF room, and a facility veterinarian is contacted immediately if a health issue is detected [11].

Spare cages are also kept in the isolator for cage changing. Co-housed animals remain as cage mates from birth to minimize aggressive behavior. Homogeneity of the GF microbiome in these mice is assured by random pairing of the co-housed mice. Animals are accessible using the port gloves, which are covered by a supplemental pair of sterile surgical gloves or cloth gloves for manipulations (Figure 2C). Colonies are age- and gender-matched, with control C57BL/6 mice housed under the same conditions in an adjacent room. All animals require one week of acclimatization before any experiments commence (Figure 2D). 

#### 3.2.2. Identification of the Mice

Ear punching was used in our model to identify the animals (for injections, fecal transplant or collection purposes). The ear punch is aseptically introduced before animals enter the isolator, with a specific code implemented to identify each animal. The control C57BL/6 mice housed in the adjacent room might have the same codes to identify the different experiments. 

#### 3.2.3. Animals Observation

Animal observation is carried out by trained technicians or investigators once daily and recorded. Cage change is performed every two weeks. Forceps are used to handle the mice to minimize a potential breach in the barrier. Isolator entries are documented on a log sheet. Each week, swabs are collected for bacterial and fungal culture testing. This includes swabs taken weekly from the port entrance walls, isolator walls, right and left gloves, mold trap, water and food stock, and cage environment. Gram stains of feces, mold trap, and feed are performed weekly, examined and compared to the previous week’s Gram stain slide to document that there are no changes in sterility. 

#### 3.2.4. Fecal Sample Collection

Prior to beginning a study, fecal samples can be collected for microbiome analysis as baseline screening, then on regular intervals to monitor any changes. Prior to autoclaving, ensure that each tube is properly labeled and/or numbered. All tubes can be placed in the isolator at the beginning of the study. (Colored tubes could reduce mistakes due to label loss/readability.) Fecal samples are collected and placed into tubes with sterile tweezers within the isolator. Following the protocol, the tubes are placed inside the SRI port and sprayed with sterilant before being removed from the isolator. Tubes are then placed in sterile pouches, sealed, and kept on dry ice to be stored at -80°C in appropriately labeled boxes. 

#### 3.2.5. Mice Injections

##### Biologic or Small Molecule Therapeutics

Sterilized 1.5 mL tubes containing therapeutics to be injected intra-peritoneally or subcutaneously can be transferred through the port entrance after being liberally sprayed with sterilant prior to injections (Figure 2B). An adequate number of individually-wrapped sterile syringes are sprayed and can be transferred en masse to cover the length of a study. Mice are removed from their cage individually for injections, then replaced and further monitored by a technician over the next few days to monitor potential adverse event occurrence. 

##### Mice Can be Fecal Transplanted (Fecal Matter Transplant, FMT) Using a Similar Protocol

Sterilized 1.5 mL tubes containing FMT preparations, (one selected strain of bacteria for example) prepared in sterile anaerobic conditions and to be given by oral gavage technique, can be transferred through the port entrance after being liberally sprayed with sterilant prior to injections. These tubes are transported in sterile sealed pouches from the sterile anaerobic chambers to the dedicated GF room and SRI sterile port entrance. An adequate number of individually-wrapped sterile syringes are sprayed and can be transferred en masse to cover the length of a study (Figure 2B). An equal number of sterile individually wrapped needles for oral gavage are transferred to the isolator following the same sterilizing protocol (Cadence Science malleable stainless-steel animal feeding needles, Fisher, Hampton, NH, USA). Mice are removed from their cage individually for oral gavage, then replaced and further monitored to ensure proper transplantation. After FMT, mice and isolators are considered gnotobiotic, but continue to be handled using sterile conditions.

#### 3.2.6. Mice Bleeding, Tissue Collection

GF mice can be bled inside the isolator by a trained investigator. Blood collection tube coding/numbering/coloring should parallel that used for fecal collection. The tubes are placed on a plastic sterile rack previously introduced and each mouse is bled. A submandibular technique, using a sterile 4 mm lancet, has proven to be an effective choice. Alternative techniques include tail bleeding or terminal bleeding. Do not perform retro-orbital blood collection in these fragile mice. From each adult mouse, blood is collected in tubes and placed into the port to be transferred outside, following the sterile protocol. It is then immediately transferred to serum tubes for sterile processing and to avoid coagulation. Similarly, collection of other tissues can be performed inside the isolator following sterile protocols.

#### 3.2.7. End of the Study

Removal of the mice from the isolator occurs under only two conditions: at the endpoint of a study, or if one of the animals is ill and needs to be euthanized per the facility Standard Operating Procedures (SOP) or as described in the animal study protocol. Control C57BL/6 mice are euthanized using similar established procedures, generally with CO_2_ or by cervical dislocation.

## 4. Expected Results and Discussion

### Characterization of phenotype (Figure 3)

Control C57BL/6 animals are housed, fed, and watered with the same autoclaved supplies as the GF colonies, including the same biofresh bedding. This step is important to avoid any bias in therapeutic studies.

**Figure 3:**
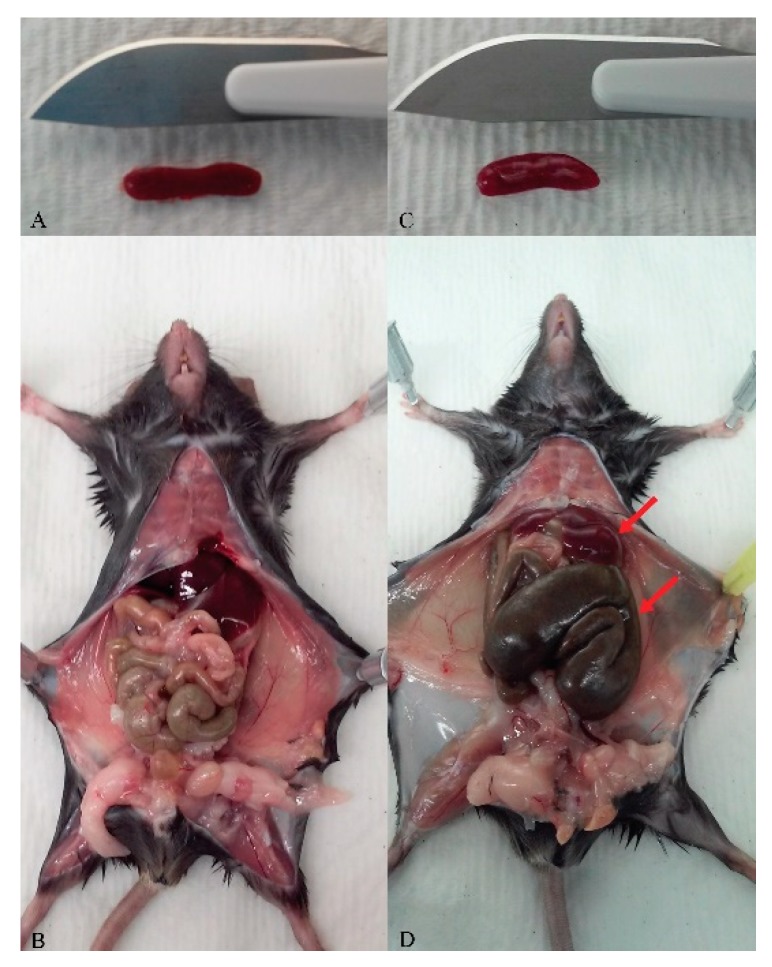
Germ-free and control mouse macroscopic phenotype. (**a**) and (**b**): Control C57BL/6 mouse, three-month-old male. Phenotype: typically, fat; normal spleen, liver, and cecum. (**c**) and (**d**): Germ-free C57BL/6 mouse, three-month-old male. Phenotype: lean, no fat; smaller spleen and liver, and enlarged cecum (5 ×). Pictures are representative of the mouse cohort and genders.

The GF mouse phenotype and description have been well documented for many years and the gastrointestinal structure and function of these animals was extensively characterized by Thompson and Trexler from London in 1971 [10]. The distended cecum, weighing about ten times more than normal, is intriguing. It has been suggested that this excess weight is due to enhanced water transport. So far, the best way to return the enlarged cecum to normal has been to colonize the gastrointestinal tract with anaerobic bacteria. The absorption, linked to the speed of transit in the small intestine, is also disturbed in GF animals; Partially digested and unabsorbed peptides accumulate in the cecum. The authors also reported that other organs, such as liver, lungs, heart, and spleen were smaller [10]. The GF mice in this protocol presented a phenotype consistent with previous publications [12,13,14]. They were lean with no discernable fat, presented a five-fold enlarged cecum relative to controls and exhibited slightly smaller organs; including smaller spleens, lungs, livers, and hearts (Figure 3A–D). Additionally, they exhibited increased heat sensitivity and an increased need for food and water. We found the animals looked very healthy and were more active than conventional mice. To our knowledge, this hyperactive phenotype had not been previously reported. 

Germ free mice have been extensively used for deciphering some mechanisms linked to diseases, such as Type 2 diabetes mellitus, T2DM [15] behavioral functions at the gut-brain axis and autism [16], cardiovascular diseases [17] and cancer [18]. The use of therapeutics to treat these diseases is also investigated with the use of germ-free colonies and use of a standardized protocol and method would be of interest for future studies.

We used this model for investigating biologic therapeutics and microbiome interactions [19], and made use of the same setup to perform fecal matter transplant (FMT) in GF mice: one isolator was dedicated to gnotobiotic mice, while the other contained GF mice. Fecal transplants were manipulated in the same way as injections and sterilely introduced into the gnotobiotic isolator before being transferred immediately to mice. They were previously prepared under sterile anaerobic conditions and introduced into the isolator in prepared syringes individually wrapped in sterile pouches. Iso cages (Tecniplast, West Chester, PA, USA) can also be used as gnotobiotic isolators, with four to six mice co-housed in each cage. 

In preclinical studies, GF animals do not mimic human conditions, but serve as models to better understand the influence of bacteria on host development and function. Moreover, colonizing the animals with one strain or a cocktail of known strains of bacteria enabled us to determine the impact of a specific bacteria on multiple health-related issues. Some alternatives to the germ-free murine model exist. The use of antibiotic treatment, probiotic feeding, fecal transplants, and mouse humanization have been explored ^8^. However, using GF animals has proven to be the best alternative so far, even though mouse models have limitations with respect to extrapolation of findings to humans.

## 5. Conclusions

We are still in the discovery phase with respect to determining the role of the gut microbiota in human health and disease. This model introduces new avenues of research facilitating extrapolation of murine findings to humans. Ascertaining the function of the large number of microorganisms colonizing human bodies, particularly in the gut, might help elucidate the onset of, and gain insight into, the progression of diseases and selection of appropriate therapies. Furthermore, our enhanced understanding of the system might enable its modulation to improve therapeutic success. The complexity of these host-bacteria interactions, the fine regulation of symbiosis in the gut, and the immune mechanisms linked to these modifications clearly present a challenge today. Deciphering the interactions between the microbiome and host cells should help address numerous questions, particularly with respect to autoimmune diseases and their therapies. Deciphering the interactions between the microbiome and host cells will help in the design of a precision medicine approach to patient treatments, in which therapeutic optimization and successful responses would be more attainable. It could also initiate the development of new therapeutics targeting the microbiome to help optimize the efficacy of biologics or small molecule drugs.
